# CircRAD23B promotes proliferation and carboplatin resistance in ovarian cancer cell lines and organoids

**DOI:** 10.1186/s12935-024-03228-1

**Published:** 2024-01-25

**Authors:** Hui Wang, Yashuang Zhang, Huixian Miao, Ting Xu, Xianglin Nie, Wenjun Cheng

**Affiliations:** Department of Gynecology, The First Affiliated Hospital of Nanjing Medical University, Nanjing, Jiangsu 210029 China

**Keywords:** CircRAD23B, Ovarian cancer, Carboplatin resistance, Patient-derived organoids, YBX1

## Abstract

**Background:**

Circular RNAs (circRNAs) are involved in the regulation of progression and drug resistance in ovarian cancer (OC). In the present study, we aimed to explore the role of circRAD23B, a newly identified circRNA, in the regulation of carboplatin-resistant OC.

**Methods:**

CircRAD23B expression levels were measured using qRT-PCR. The biological roles of circRAD23B were analysed using CCK-8, colony formation, EDU, flow cytometry, and cell viability assays. RNA pull-down and luciferase assays were used to investigate the interactions of circRAD23B with mRNAs and miRNAs.

**Results:**

CircRAD23B was significantly increased in carboplatin-resistant OC tissues. CircRAD23B promoted proliferation and reduced sensitivity to carboplatin in cell lines and patient-derived organoids (PDOs), consistent with in vivo findings. Mechanistically, circRAD23B acted as a molecular sponge, abrogating its inhibitory effect on Y-box binding protein 1 (YBX1) by adsorbing miR-1287-5p. Rescue experiments confirmed that the pro-proliferation and carboplatin resistance mediated by circRAD23B was partially reversed by the upregulation of miR-1287-5p.

**Conclusions:**

Our results demonstrated, for the first time, the role of the circRAD23B/miR-1287-5p/YBX1 axis in OC progression and carboplatin resistance in cell lines, PDOs, and animal models, providing a basis for the development of targeted therapies for patients with OC.

**Supplementary Information:**

The online version contains supplementary material available at 10.1186/s12935-024-03228-1.

## Background

Ovarian cancer (OC) is the most lethal malignancy in women, with a 5-year survival rate of 30% [1]. Most patients are diagnosed with advanced OC at their first visit, as patients with early stage OC have no obvious symptoms. Maximal cytoreductive surgery followed by platinum-based combination chemotherapy is the standard treatment for OC. Although more than 80% of patients achieve complete remission after the standard treatment, recurrence within 2 years owing to chemoresistance is common and severely affects their prognosis [2]. Therefore, the mechanism of platinum resistance in OC warrants exploration and is essential for the discovery of reliable diagnostic markers and development of new treatments to overcome chemoresistance.

The role of non-coding RNAs (ncRNAs) in OC has gradually been revealed in recent years [3]. miRNAs and long non-coding RNAs (lncRNAs) play important roles in promoting OC malignancy and inducing platinum resistance [4–6]. Research on circular RNAs(circRNAs), a newly discovered ncRNA type, is still in its infancy in terms of its association with tumour proliferation and platinum resistance [7–9]. CircRNAs are linked by a covalent bond to form a closed loop structure which is highly insensitive to nucleases and has strong stability [10]. Increasing evidence has reported that circRNAs play an important role in regulating gene expression at transcriptional and post-transcriptional levels [11]. The main established mechanisms include sponging of miRNAs [12], transcriptional regulation [13], scaffolding or sponging of proteins [14], interaction with mRNAs [15] and translation of proteins [16]. Among them, miRNA sponges are widely believed to be the dominant regulators.

Recently, the functions of circRNAs in cancer development and progression have attracted increasing attention. Evidence has demonstrated that many circRNAs contribute to cancer hallmarks such as limitless proliferation, tissue invasion and metastasis, sustained angiogenesis and tumour-promoting inflammation [17, 18]. Some circRNAs have been shown to regulate OC cell proliferation [19], metabolism [20], epithelial-mesenchymal transition [21] and drug resistance [22]; thus, they are thought to be potential biomarkers or therapeutic targets [23, 24]. However, the effect of circRNAs on OC progression and drug resistance remains unclear.

Among the different genes of circRNAs, *RAD23B* is a notable one in cancer research. It is the parental gene of hsa_circ_0087855 and hsa_circ_0087862 and is highly expressed in a variety of tumours and contributes to the malignant biological phenotype [25–27]. Although *RAD23B* is known to promote tumorigenesis and progression in non-small cell lung [28], oesophageal [29] and colorectal cancers [30], its function in OC remains unknown. In this study, we aimed to investigate the role of circRAD23B in OC.

## Methods

### Clinical sample collections

OC tissues from 76 cases were resected from Jiangsu Provincial People’s Hospital (the First Affiliated Hospital of Nanjing Medical University), within the Department of Gynaecology. Tissues were frozen in liquid nitrogen and stored in a freezer at -80 °C. None of the patients in this study received OC-related treatment before surgery. Patients who relapsed within 12 months after the end of first-line chemotherapy were assigned to the drug-resistant group, and those who relapsed after > 12 months were assigned to the sensitive group. All participants provided written informed consent, and the study was approved by the Ethics Committee of Jiangsu Provincial People’s Hospital. Correlations between the clinicopathological characteristics of the 76 patients with OC are shown in Table 1.

**Table 1:** The relationship between carboplatin resistance and clinicopathologic features of patients with OC

Characteristics	Resistant	Sensitive	P value
n	42	34	
Age, mean ± SD	58.905 ± 7.0635	59.941 ± 5.5919	0.488
circRAD23B expression, median (IQR)	0.055635 (0.031743, 0.068631)	0.034036 (0.010424, 0.050689)	0.008
Stage, n (%)			0.659
Stage2	5 (6.6%)	2 (2.6%)	
Stage3	26 (34.2%)	22 (28.9%)	
Stage4	11 (14.5%)	10 (13.2%)	
Patients’ weight, median (IQR)(Kg)	51 (47, 62)	56.5 (52.25, 63.5)	0.136

### Organoid preparation

The procedure for preparing patient-derived organoids (PDOs) was based on a previously reported protocol [31]. Fresh tumour tissue samples were sliced into 1–2 mm slices, blood was rinsed with ice-cold PBS^CaMg free^, and as much fat and necrotic tissue were removed as possible. Appropriate tissue-embedded frozen sections were collected, and the rest of the samples were digested with DNase II (D-4693, Sigma, MA) at 37 °C for 30 min. The supernatant was collected after discarding the undigested tissue pieces, filtered through a 40 μm cell sieve, and single cells were washed with washing buffer to obtain organoid glands. Primary organoids were embedded in 70% Matrigel, and growth medium was added to 48-well plates. Organoids were replaced with fresh medium every three days and passaged every to 2–3 weeks.

### Cell culture

Human OC cells (Ovcar3, HO8910, A2780, HEY) and normal ovarian epithelial cells were cultured in RPMI − 1640 medium with 10% foetal bovine serum (Gibco, Grand Island, NY, USA) and 1% penicillin/streptomycin (Procell, Wuhan, China) at 37 °C in a 5% CO_2_ incubator.

### RNA extraction and qRT-PCR

Total RNA was extracted from OC tissues and cells using an animal RNA isolation kit (Beyotime, Shanghai, China) according to the manufacturer’s protocols. The concentration and quality of the RNA were detected using a NanoDrop spectrophotometer (ND-100, Thermo). cDNA was prepared using the HiScript Q RT SuperMix for qPCR (Vazyme, China). miRNAs were reverse transcribed using the miRNA 1st Strand cDNA Synthesis Kit (stem-loop) MR101 (Vazyme, China). qRT-PCR was performed using ChamQ Universal SYBR qPCR Master Mix Q711 (Vazyme, China). The following primer sequences were used:

CircRAD23B (divergent primers).

Forward primer 5ʹ ACACCTGCATCCATCACTCC 3ʹ.

Reverse primer 5ʹ AGTGATGGATGCAGGTGTGG 3ʹ.

CircRAD23B (convergent primers).

Forward primer 5ʹ ACAACTCAGCAGTCAGCTCC 3ʹ.

Reverse primer 5ʹ AGTGATGGATGCAGGTGTGG 3ʹ.

miR-1287-5p.

Forward primer 5ʹ GCGGTGCTGGATCAGTGG 3ʹ.

Reverse primer 5ʹ CAGTGCAGGGTCCGAGGTAT 3ʹ.

U6.

Forward primer 5ʹ GCTTCGGCAGCACATATACTAAAAT 3ʹ.

Reverse primer 5ʹ CGCTTCACGAATTTGCGTGTCAT 3ʹ.

GAPDH.

Forward primer 5ʹ TATGATGACATCAAGAAGGTGGT 3ʹ.

Reverse primer 5ʹ TGTAGCCAAATTCGTTGTCATAC 3ʹ.

YBX1.

Forward primer 5ʹ TAGACGCTATCCACGTCGTAG 3ʹ.

Reverse primer 5ʹ ATCCCTCGTTCTTTTCCCCAC 3ʹ.

### Agarose gel electrophoresis and RNA stability test

Circular and linear RAD23B transcripts were amplified using divergent and convergent primers for OC cDNA and gDNA, respectively. The PCR products were subjected to agarose gel electrophoresis. OC cells were treated with 1 mg/mL actinomycin D solution (MCE, New Jersey, USA). Cells were collected at specific time points (0, 3, 6, and 9 h). Total RNA was extracted from OC cells, treated with RNase R, and incubated for 15 min at 37 °C. Subsequently, qRT-PCR was performed to detect the expression of RAD23B and circRAD23B.

### Lentiviral, plasmid, and oligonucleotide transfection

Lentiviral vectors containing pLV-circRAD23B, sh-circRAD23B, or negative controls were purchased from ViGene Biosciences (Shandong, China). MiR-1287-5p mimic and biotin-miR-1287-5p probes were obtained from RiboBio (Guangzhou, China). Transfection was performed using Lipofectamine 3000 (Invitrogen) according to the manufacturer’s instructions. After 48 h of transfection, real-time PCR was performed to determine the transfection efficiency in each group of cells. Stable cell lines and PDOs were obtained via puromycin screening.

### Western blotting

Proteins were extracted using a lysis buffer, separated by SDS-polyacrylamide gel electrophoresis and transferred onto PVDF membranes (Thermo Fisher Scientific, Waltham, MA, USA). Primary antibodies were hybridised overnight at 4 °C, and secondary antibodies were immunoblotted to obtain blot images. Primary antibodies against YBX1 (A7704, Abclonal) and GAPDH (AC001, Abclonal) were used.

### RNA fluorescence in situ hybridization (FISH)

FISH was performed according to the manufacturer’s instructions. Cy3-labelled circRAD23B probe and Fam-labelled miR-1287-5p were designed and synthesised by RiboBio (Guangzhou, China). Signal detection and image acquisition were achieved using a Leica SP5 confocal microscope (Leica Microsystems).

### RNA pull-down

Biotin-labelled miR-1287-5p and control probes were obtained from RiboBio (Guangzhou, China). The biotinylated miRNA probe was mixed with Dynabeads M-280 Streptavidin (Thermo Fisher Scientific) for 2 h at room temperature. OC cells were lysed in 150–200 µL of RIPA buffer at 0 °C for 20 min, and then incubated with probe-coated beads overnight at 4 °C. The cells were then washed with lysis buffer. Finally, the bound RNA was purified, and qRT-PCR was performed to detect the abundance of circRAD23B and YBX1.

### Colony formation, cell proliferation and EDU assays

Five hundred cells per plate were analysed for colony formation in 6-well plates. Two weeks later, cells were stained with 0.1% crystal violet and fixed in ethanol for 30 min. Cell colonies were counted and analysed. Cell proliferation was measured using the CCK-8 assay (Vazyme, China). Fifteen hundred cells were inoculated into 96-well plates, and 10 µL CCK-8 solution was added to each well and incubated with the cells for 2 h. Cell numbers were determined at specific time points (days 1, 2, 3, 4, and 5) by measuring the absorbance (450 nm). EDU assays were performed using the Cell-Light EDU DNA Cell Proliferation Kit (RiboBio, Guangzhou, China). Ten thousand cells were seeded into each well of a 96-well plate. Cells were incubated in 50 µM EDU solution for 2 h and then fixed in 4% paraformaldehyde and stained with Apollo staining solution. The nucleic acids inside the cells were stained with Hoechst 33,342. Images were obtained using a microscope (Olympus, Tokyo, Japan).

### Flow cytometry

HO8910 and A2780 were treated with 20 µM carboplatin, and cells were collected after 48 h and rinsed twice with PBS^CaMg free^. Cells were stained using the Annexin V-FITC/propidium iodide (PI) Apoptosis Detection Kit (#556,547; BD Biosciences) according to the manufacturer’s protocol, and apoptotic cells were detected using flow cytometry.

### Cell viability

After 48 h incubation of organoids in 48-well plates, carboplatin 1-100 µM was added to the organoids for another 2 days. CellTiter-Glo® Luminescent Cell Viability Assay (G7570, Promega, Wisconsin) was used to detect cell viability. The IC_50_ values were calculated for several organoids after assessing their viability using an enzyme marker. Organoids were stained using a cyto3D Live-Dead Assay kit (BM01, Well Bioscience, NJ, USA). Live and dead cells were labelled green and red, respectively, and photographed using a fluorescence microscope.

### Haematoxylin-eosin (HE) and immunohistochemical (IHC) staining

The organoids were washed with ice-cold PBS^CaMg free^, digested with cell recovery solution (354,253, Corning, NY), and centrifuged. After fixing with 4% paraformaldehyde (P0099; Beyotime, Shanghai, China), the organoids were dehydrated in a 30% sucrose solution for 24 h and embedded in O.C.T ice gel (4583; Solarbio, Beijing, China) for sectioning. The sections were treated with xylene and graded ethanol, followed by antigen repair with citrate buffer (P0081; Beyotime), organoid permeabilization, and blocking. The nuclei were stained with DAPI (C1002, Beyotime) after addition of the corresponding primary antibody and fluorescently labelled secondary antibody. After fixing the slides with neutral resin, they were photographed and observed under a fluorescence microscope. Primary antibodies against PANCK (26411-1-AP, Proteintech), PAX8 (10336-1-AP, Proteintech), WT-1(A2446, ABclonal), P53 (A0263, ABclonal), and Ki67 (A20018, ABclonal) were used. HE and IHC staining were performed using Servicebio (Wuhan, China).

### Mouse xenograft model

All experiments were approved by the Animal Care and Use Committee of the Jiangsu Provincial People’s Hospital. The Declaration of Helsinki was used as a principle for animal research. Female BALB/c nude mice of age 5 weeks were purchased from the Department of the Experimental Animal Centre of Nanjing Medical University. We subcutaneously injected 5 × 10^6^ transfected cells with 100 µL into nude mice. Detection tests for subcutaneous tumours were performed once per week and collected after four weeks. Tumour size was measured regularly with digital callipers, and the formula for tumour volume detection was V = 0.5 × length × width^2^.

### RNA sequencing

Total RNA was extracted from cells using an RNA extraction kit (RC101-01; Vazyme, China). Libraries were generated using an RNA library preparation kit (E7530L, NEB, NY, USA) and sequenced on the PE150 platform (UW Genetics, Shenzhen, China). After downloading and cleaning the raw data, sequences were compared using the HISAT2 package, and differential gene analysis was performed using the Deseq2 package with a threshold of *P* < 0.05 and |log_2_FC|>1 for the screening of genes for further analysis.

### Statistical analysis

Data were expressed as mean ± standard deviation (SD). Clinicopathological results were analysed using an unpaired *t*-test or Pearson χ2 test. Differences between groups were analysed using Student’s *t*-test or one-way ANOVA. All statistical analyses were performed using SPSS (version 20.0) (IBM, Armonk, NY, USA) and GraphPad Prism (version 7.0). P values less than 0.05 were considered statistically significant.

## Results

### CircRAD23B was upregulated in OC tissues and cells

**Fig. 1:**
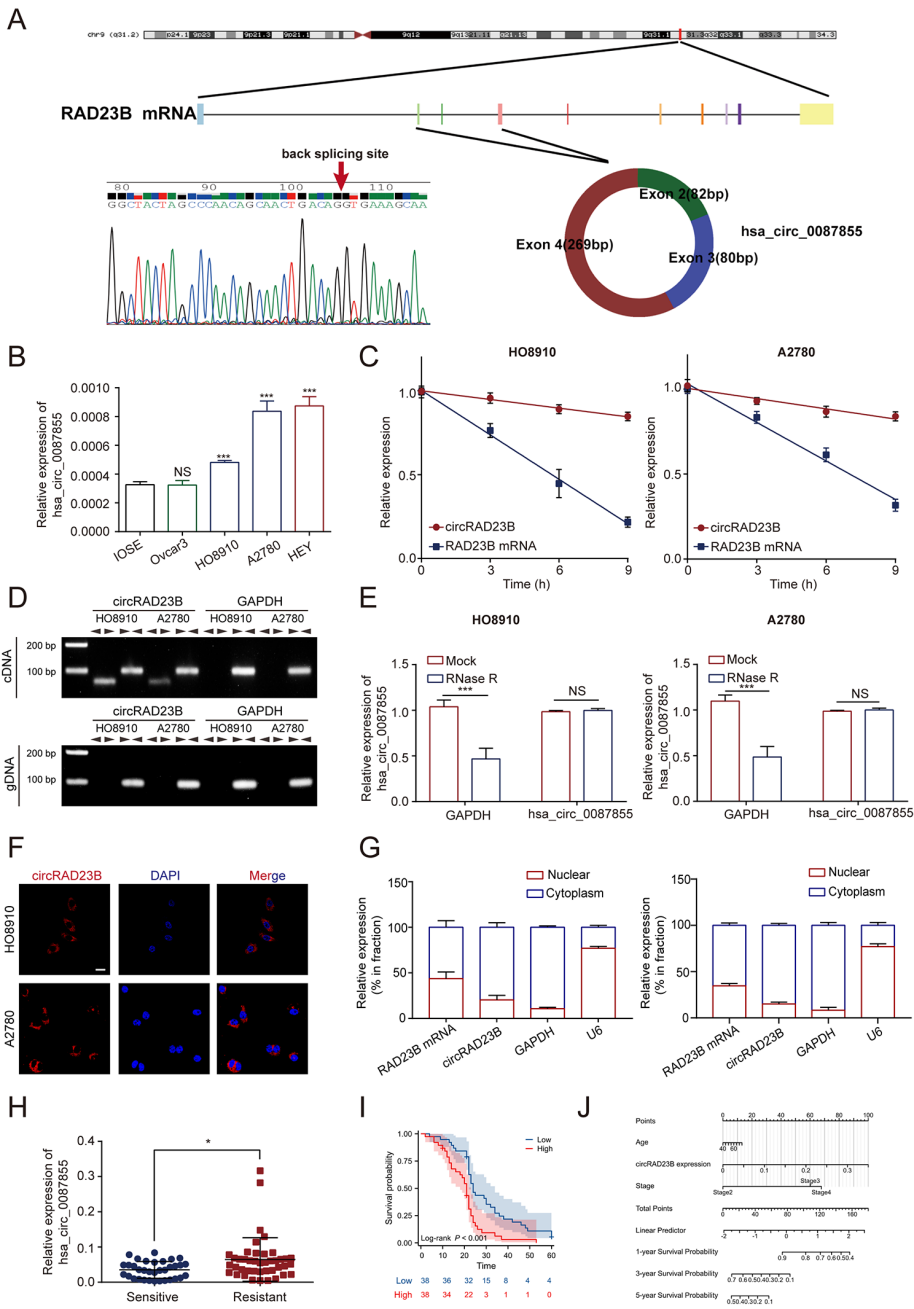
Characteristics of circRAD23B in ovarian cancer. (**A**) The schematic diagram of circRAD23B (hsa-circ-0087855) arose from exon 2,3,4 of the RAD23B gene. The sequence of hsa-circ-0087855 in circBase was consistent with the result of Sanger sequencing. (**B**) The expression level of circRAD23B in different ovarian cancer cells. (**C**) The RNA level of circRAD23B and RAD23B was examed at four time pionts after the treament of Actinomycin D in HO8910 and A2780 cell lines. (**D**) The divergent primers detected circRAD23B in cDNA but not in gDNA, GAPDH was used as a negative control. (**E**) The stability of circRAD23B was not affected by RNase R in HO8910 and A2780 cell lines. (**F**) Immunofluorescence assay showed that circRAD23B was localized in the cytoplasm. (**G**) RNA nucleoplasmic separation assay showed that circRAD23B was mainly distributed in the cytoplasm, and U6 and GAPDH were used as positive controls for the nucleus and cytoplasm, respectively. (**H**) Relative expression of circRAD23B in carboplatin-sensitive and -resistant patients. (**I**) The overall survival analysis in patients with different circRAD23B level. (**J**) Nomogram containing common clinicopathological factors and circRAD23B expression in ovarian cancer patients. Graph represents mean ± SD; *p < 0.05, and ***p < 0.001

Sanger sequencing was performed to determine the characteristics of circRAD23B. CircRAD23B (circBase ID: hsa_circ_0087855) was derived from exons to 2–4 of *RAD23B* and formed a full-length circular transcript of 431 bp, consistent with the results of Sanger sequencing **(**Fig. 1A**)**. qRT-PCR confirmed that circRAD23B was upregulated in HO8910, A2780, and HEY cells compared to its expression in the normal cells (Fig. 1B). In addition, actinomycin D treatment did not impact the half-life of circRAD23B as much it impacted that of RAD23B mRNA (Fig. 1C). Next, divergent and convergent primers were designed to identify the properties of circRAD23B (Fig. 1D). CircRAD23B was shown to have a nonlinear structure because RNase R did not affect its expression (Fig. 1E) and was preferentially expressed in the cytoplasm, as indicated by FISH (Fig. 1F) and nuclear extraction assays (Fig. 1G). To further evaluate the clinical value of circRAD23B, we examined circRAD23B expression in OC samples. CircRAD23B was significantly increased in the carboplatin-resistant group (Fig. 1H) and negatively correlated with the overall survival of patients with OC (Fig. 1I). Finally, we constructed a clinical prediction model based on the expression of circRAD23B, patients’ age, and OC stage to predict the prognosis of patients with OC (Fig. 1J).

### CircRAD23B promoted the progression of OC and induced carboplatin resistance in cells

**Fig. 2:**
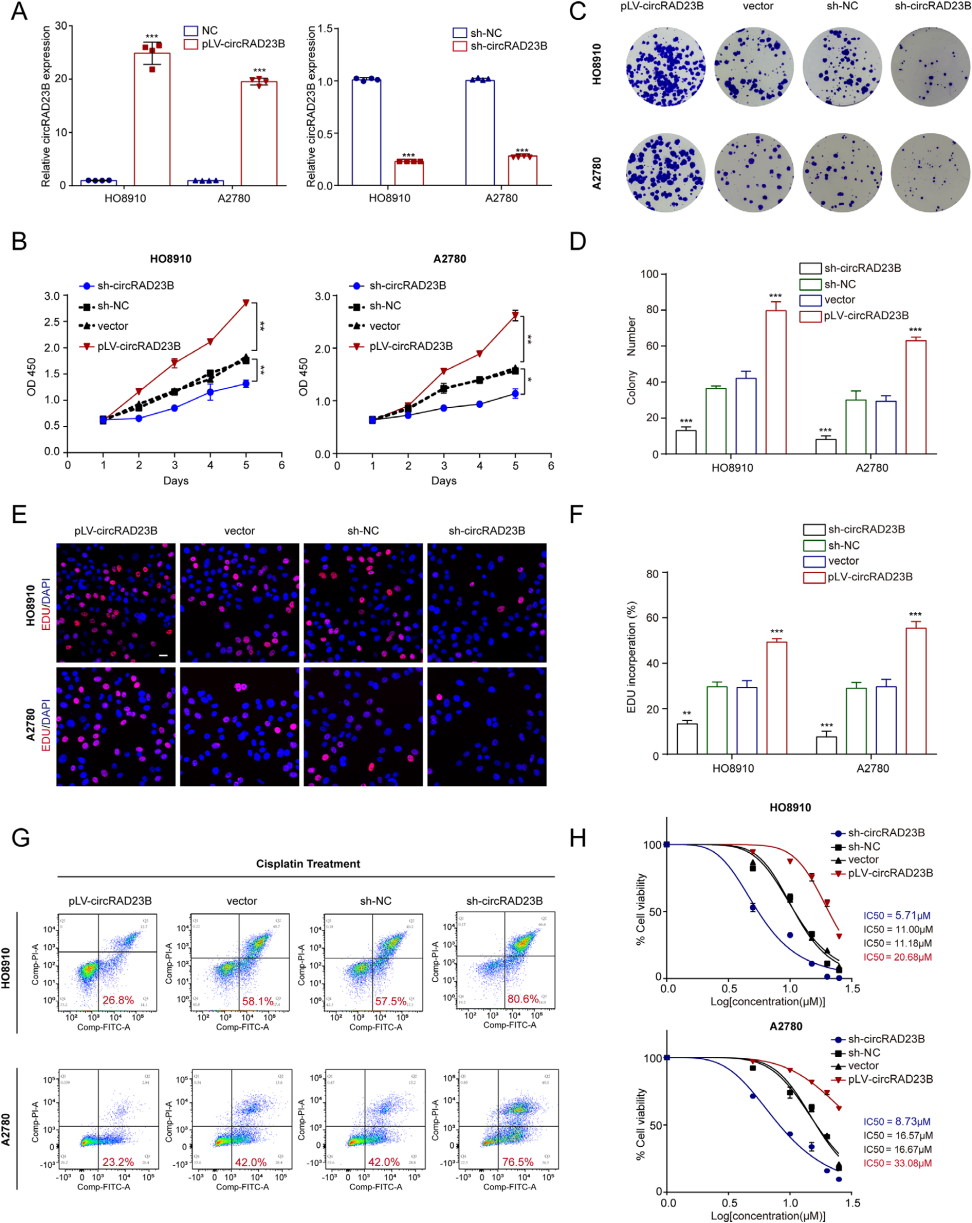
Functions of circRAD23B in ovarian cancer cells. (**A**) qRT-PCR was used to verify the successfully establishment of circRAD23B overexpression and knockdown cell lines. (**B**) CCK-8 assay in HO8910 and A2780 cells transfected with circRAD23B shRNAs or pLV-circRAD23B. (**C**) Representative images of colony formation. (**D**) Statistical analysis of colony formation. (**E**) Representative images of EDU exprements. (**F**) Statistical analysis of EDU exprements. Red represents EDU staining, blue represents DAPI staining. (**G**) Apopotosis rate was analysed by flow cytometry after carboplatin treatment. (**H**) The IC_50_ of carboplatin in HO8910 and A2780 cells transfected with circRAD23B shRNAs or pLV-circRAD23B. Graph represents mean ± SD; *p < 0.05, **p < 0.01, and ***p < 0.001

To identify the role of circRAD23B in OC, pLV-circRAD23B and sh-circRAD23B cells were transfected with HO8910 and A2780 cells (Fig. 2A). Upon overexpression or knockdown of circRAD23B, the proliferative capacity of HO8910 and A2780 cells increased or decreased, respectively (Fig. 2B). The size and number of clones decreased after circRAD23B knockdown, whereas the opposite trend was observed after circRAD23B overexpression (Fig. 2C,D). EDU experiments further confirmed that overexpression of circRAD23B accelerated tumour growth, whereas knockdown of circRAD23B inhibited tumour growth in OC cells (Fig. 2E,F). To investigate the relationship between circRAD23B and carboplatin resistance in OC, we measured cell apoptosis by flow cytometry after 48 h of treatment with 20 µM carboplatin. As expected, the proportion of apoptotic cells in pLV-circRAD23B-transfected cells was notably decreased, whereas that in sh-circRAD23B-transfected cells was markedly increased (Fig. 2G). The IC_50_ value of carboplatin in sh-circRAD23B cells was much lower than that in pLV-circRAD23B cells, which was consistent with the flow cytometry findings (Fig. 2H).

### CircRAD23B promoted the progression of OC and induced carboplatin resistance in PDOs

**Fig. 3:**
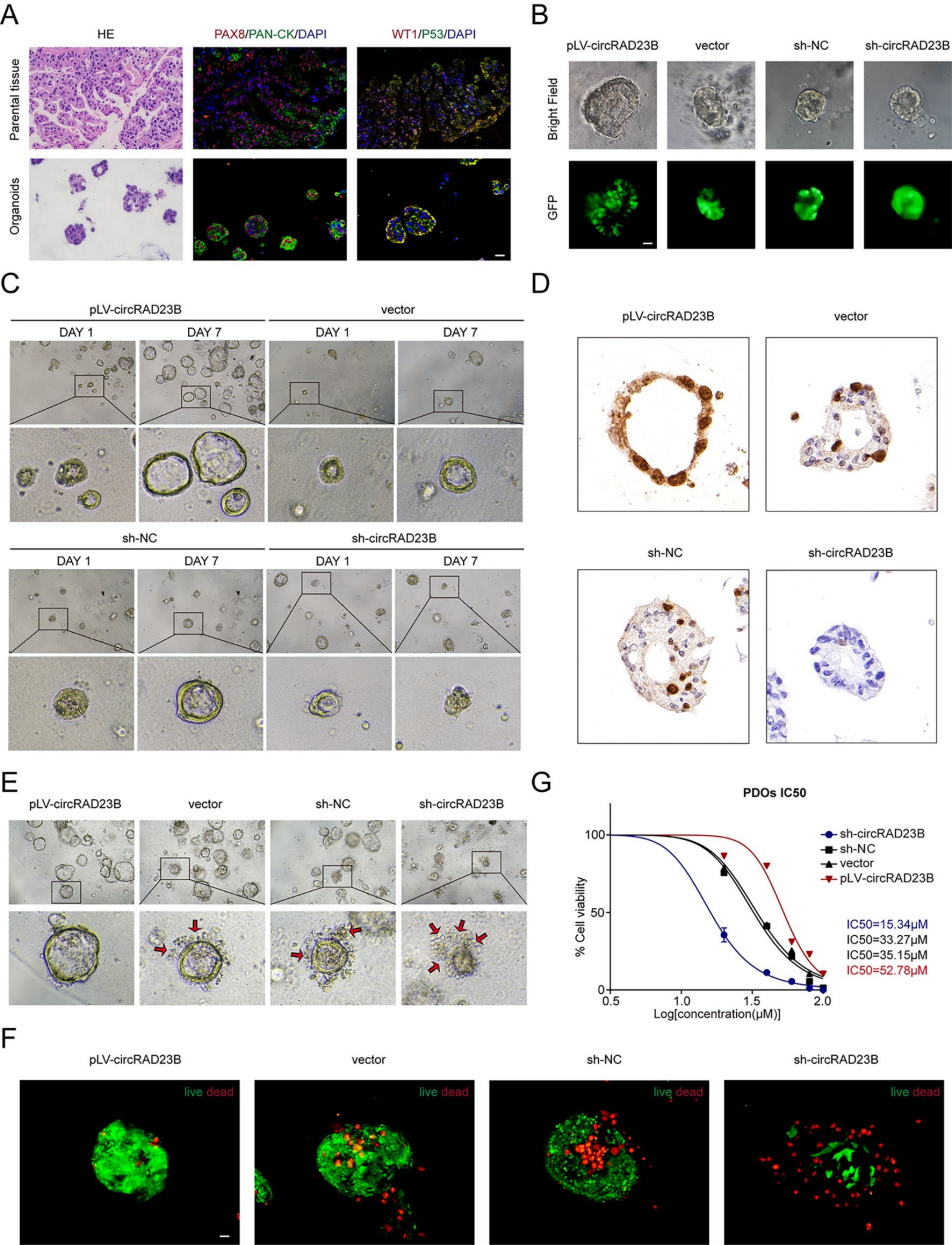
Functions of circRAD23B in patient-derived organoids (PDOs). (**A**) Immunofluorescence staining of primary tumours and PDOs, scale = 25 μm. (**B**) Organoids were transfected with sh-circRAD23B(or NC) and pLV-circRAD23B(or vector). (**C**) The size of organoids was observed in brightfield at different time pionts, scale = 25 μm. (**D**) Immunohistochemistry of Ki67 in PDOs. (**E**) Morphological changes of PDOs under 40 µM carboplatin pressure for 48 h. The red arrow indicates apoptotic cells, scale = 25 μm. (**F**) Organoids stained for cell viability/death; live cells are green, while dead cells are red. (**G**) IC_50_ values were determined to assess sensitivity of various organoids to carboplatin

To better simulate tumour growth in vivo, we established PDOs from fresh ovarian tumour tissue. Immunofluorescence (IF) of the molecular markers PAX8/PANCK/WT-1/P53 and HE staining verified that PDOs recapitulated the heterogeneity and histological phenotype of the primary tissues (Fig. 3A). The GFP signal indicated that the lentiviruses were successfully transfected into organoids to control the expression of circ-RAD23B (Fig. 3B). After 7 days, the proliferation rate was greatly increased in pLV-circRAD23B transfected PDOs but was reduced in sh-circRAD23B transfected PDOs (Fig. 3C). Immunohistochemistry of Ki67 in PDOs also demonstrated that pLV-circRAD23B transfected PDOs exhibited greater proliferative capacity than sh-circRAD23B transfected PDOs (Fig. 3D). After adding 40 µM carboplatin for 48 h in PDOs, the sh-circRAD23B transfected PDOs underwent significant disintegration and a large number of apoptotic cells were observed in the stromal gel, while the pLV-circRAD23B transfected PDOs were able to maintain the complete cystic structure (Fig. 3E). As detected by red fluorescence, apoptotic cells were more in sh-circRAD23B transfected PDOs than in pLV-circRAD23B transfected PDOs (Fig. 3F). The IC_50_ value of sh-circRAD23B transfected PDOs was lower than that of pLV-circRAD23B transfected PDOs (Fig. 3G). Overall, circRAD23B was shown to enhance carboplatin resistance in PDOs, and knockdown of circRAD23B increased the sensitivity of PDOs to carboplatin.

### CircRAD23B acted as a sponge of miR-1287-5p to regulate YBX1 expression

**Fig. 4:**
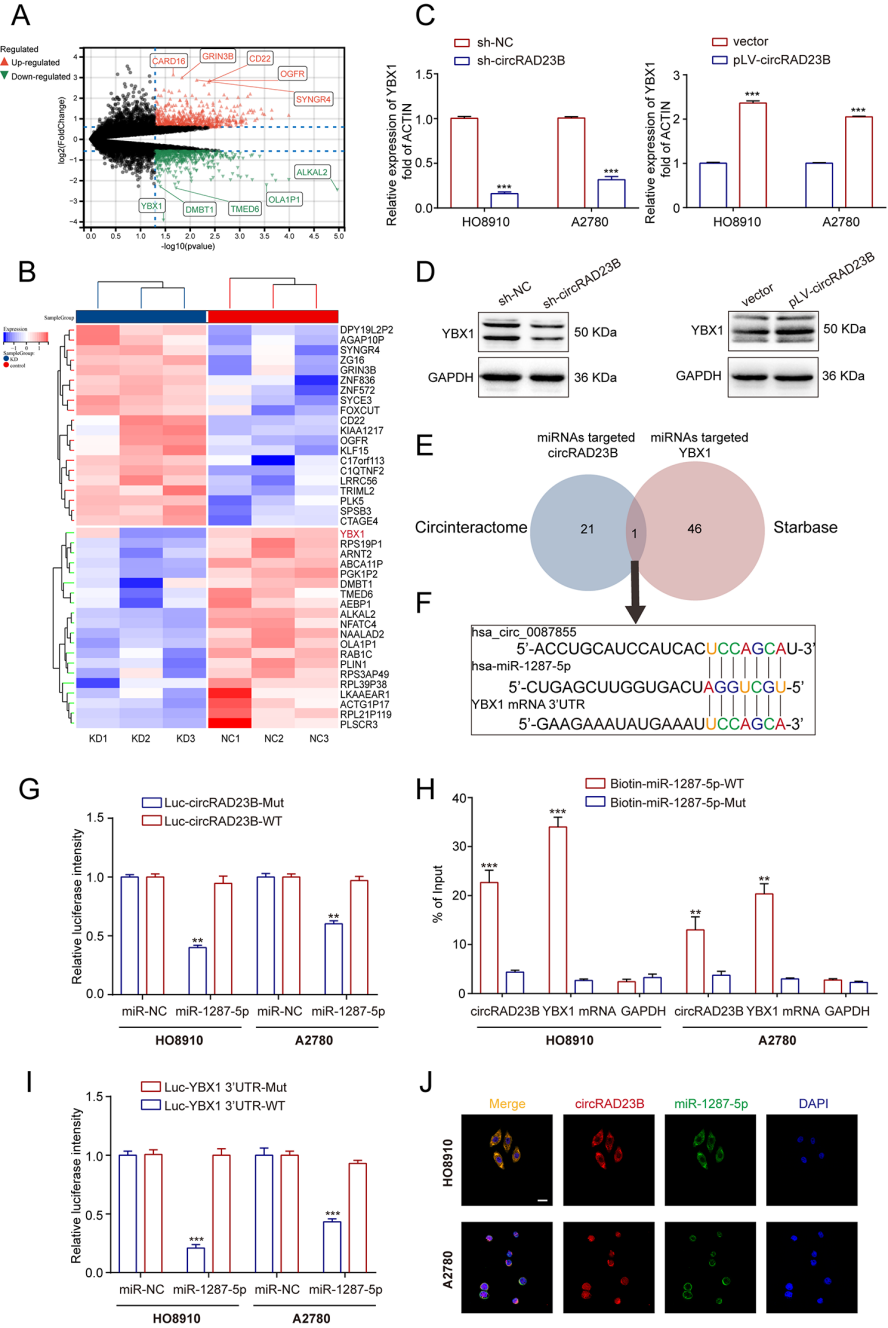
CircRAD23B acted as a sponge to regulate YBX1 expression. (**A**) Volcano map displaying upregulated and downregulated genes after knock down circRAD23B. (**B**) Heat plot showing the differentially expressed genes between sh-NC and sh-circRAD23B groups. (**C**, **D**) qRT-PCR and western blotting assay were performed to detect expression of YBX1. (**E**) Venn diagram showing the overlap of downstream miRNAs of circRAD23B predicted by the CircInteractome database and miRNAs predicted by starBase to regulate YBX1. (**F**) The binding sequence of miR-1287-5p to YBX1 mRNA and circRAD23B. (**G**) Luciferase intensity in HO8910 and A2780 cells co-transfected with luciferase reporter containing with wild-type or mutated circRAD23B-miR-1287-5p binding sequences and the mimics of miR-1287-5p or control. (**H**) The expression levels of circRAD23B and YBX1 mRNA were tested by qRT-PCR after pull-down with biotin-labeled wild-type or mutant miR-1287-5p in HO8910 and A2780 cells. (**I**) Luciferase intensity in HO8910 and A2780 cells co-transfected with luciferase reporter containing with wild-type or mutated YBX1 3’UTR-miR-1287-5p binding sequences and the mimics of miR-1287-5p or control. (**J**) Immunofluorescence assay for elaborating the relationship between circRAD23B and miR - 1287-5p, scale = 25 μm. Graph represents mean ± SD; **p < 0.01, and ***p < 0.001

Querying the circRNADb database revealed that circRAD23B does not encode a protein [32]. To determine the pathway through which circRAD23B promotes OC cell proliferation and drug resistance, we performed RNA-seq (Fig. 4A). After circRAD23B knockdown, 179 and 132 genes were upregulated and downregulated, respectively, in HO8910 cells (|log2foldchange|>1,*P*<0.05; Table S1). Among the downregulated genes, YBX1 ranked the highest (Fig. 4B). Knockdown or overexpression of circRAD23B resulted in the corresponding downregulation or up-regulation of mRNA and protein levels of YBX1 (Fig. 4C,D), which confirmed our hypothesis. Combining the CircInteractome [33] and starBase databases [34], We found that circRAD23B may regulate YBX1 by binding to miR-1287-5p (Fig. 4E). The binding sites of circRAD23B, miR-1287-5p, and YBX1 are shown in Fig. 4F. Luciferase reporter assays were performed to validate the direct binding of circRAD23B to miR-1287-5p. Overexpression of miR-1287-5p in HO8910 and A2780 cells greatly reduced the luciferase activity of the reporter gene containing the wild-type circRAD23B sequence but had no effect on the luciferase activity of the circRAD23B reporter gene containing the mutant miR-1287-5p binding site (Fig. 4G). In addition, compared to the biotin-labelled mutant miR-1287-5p, wild-type miR-1287-5p captured more circRAD23B and YBX1 mRNA in circRAD23B overexpressing OC cells (Fig. 4H), indicating that miR-1287-5p binds to circRAD2B or YBX1 mRNA. Furthermore, a luciferase reporter gene assay revealed the direct binding of miR-1287-5p and YBX1 mRNA (Fig. 4I), and the FISH assay showed the co-localisation of circRAD23B and miR-1287-5p in the cytoplasm (Fig. 4J).

### CircRAD23B/miR-1287-5p/YBX1 axis regulated proliferation and carboplatin resistance of OC

**Fig. 5:**
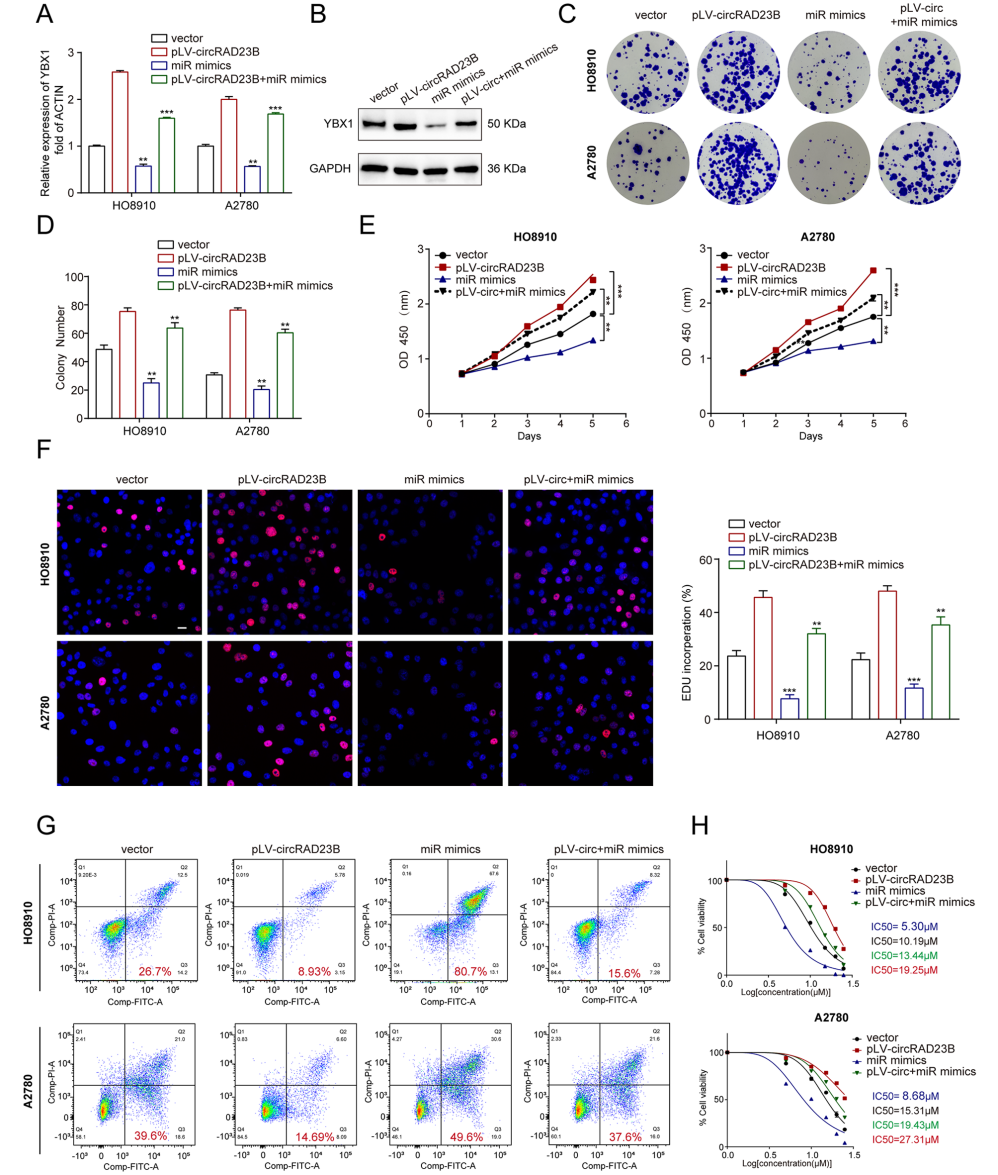
CircRAD23B/miR-1287-5p/YBX1 axis regulated proliferation and carboplatin resistance of ovarian cancer. (**A**) The expression of YBX1 mRNA in different transfected ovarian cancer cell lines. (**B**) The expression of YBX1 protein in different transfected ovarian cancer cell lines. (**C**) Representative images of colony formation. (**D**) Statistical analysis of colony formation. (**E**) CCK-8 assay in HO8910 and A2780 cells transfected with pLV-circRAD23B, vector, miR-1287-5p mimics and pLV-circRAD23B + miR-1287-5p mimics. (**F**) Representative images of EDU exprements and statistical analysis of EDU exprements. Red represents EDU staining, blue represents DAPI staining. (**G**) Apopotosis rate was analysed by flow cytometry after carboplatin treatment. (**H**) The IC_50_ of carboplatin in HO8910 and A2780 cells transfected with pLV-circRAD23B, vector, miR-1287-5p mimics and pLV-circRAD23B + miR-1287-5p mimics. Graph represents mean ± SD; **p < 0.01, and ***p < 0.001

Because miR-1287-5p binds directly to circRAD23B and YBX1 mRNA, we next explored their regulatory relationships. Through qRT-PCR and western blotting, the expression of YBX1 was found to be reduced when miR-1287-5p was overexpressed but could be rescued after circRAD23B overexpression (Fig. 5A,B). Further in vitro functional assays confirmed that miR-1287-5p overexpression inhibited tumour proliferation, and this effect was diminished by the overexpression of circRAD23B (Fig. 5C-F). Flow cytometry and IC_50_ values suggested that the miR-1287-5p mimic enhanced carboplatin sensitivity, whereas pLV-circRAD23B partially attenuated carboplatin sensitivity in OC (Fig. 5G, H). Our results demonstrate the function of the circRAD23B/miR-1287-5p/YBX1 axis in the development and chemoresistance of OC.

### CircRAD23B promoted tumour development in vivo

**Fig. 6:**
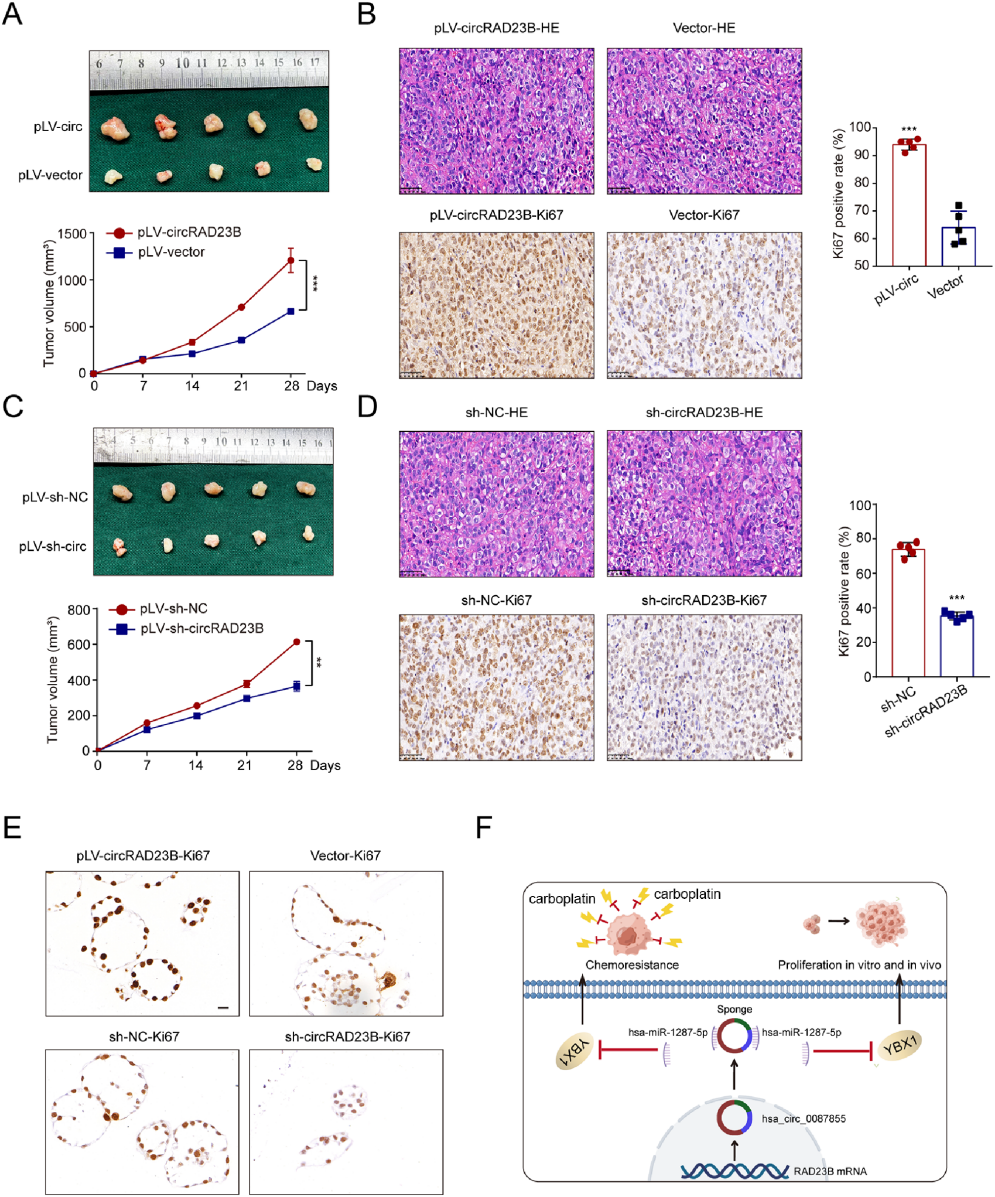
Effect of circRAD23B on tumour growth in vivo. (**A**, **C**) Excised tumours were collected at specified time pionts. (**B**, **D**) HE staining and immunochemistry of Ki67 in xenografted tumours. (**E**) Ki67 level of organoids derived from xenografted tumours. (**F**) The pattern diagram of circRAD23B /miR-1287-5p/YBX1 axis in the progression and chemoresistance of ovarian cancer. Graph represents mean ± SD; **p < 0.01, and ***p < 0.001

Finally, we evaluated the effect of circRAD23B on tumorigenesis of OC cells in nude mice. Tumour cells transfected with either pLV-circRAD23B or sh-circRAD23B were transplanted into mice and collected on day 28. Consistent with our in vitro findings, tumours transfected with pLV-circRAD23B grew much faster than those transfected with sh-circRAD23B (Fig. 6A,C). Immunohistochemistry showed that the fraction of Ki67 positive nuclei was increased in pLV-circRAD23B tumours and decreased in circRAD23B knockdown tumours (Fig. 6B,D). Organoids were derived from xenograft tumours and cultured in vitro. Overexpression or knockdown of circRAD23B in xenograft-derived organoids also resulted in enhanced or diminished proliferative capacity, respectively (Fig. 6E). The graphical summary of this study is shown in Fig. 6F.

## Discussion

In the present study, circRAD23B was found to be stably expressed in the cytoplasm of OC cells, markedly increased in drug-resistant tissues and associated with poor prognosis. Further, in vitro functional assays demonstrated that circRAD23B promotes proliferation and enhances carboplatin resistance in OC cells. Overexpression or knockdown of circRAD23B promotes or attenuates tumour proliferation, respectively, in vivo. Overall, circRAD23B was identified as a novel therapeutic target for OC treatment.

Notably, we employed a PDO model to evaluate circRNA functions in OC. Patient-derived organoids have been one of the extraordinary achievements of 3D cellular models in the past decades and could recapitulate the biological behaviour of primary tissues in many cancers, especially in terms of drug sensitivity [35]. In this study, we established OC carcinoids using freshly resected OC tumour tissues. CircRAD23B was knocked down or overexpressed by lentiviral transfection in OC carcinoids and promoted OC carcinoid proliferation and chemoresistance. To our knowledge, this is the second study exploring the functions of circRNAs in an organoid model. Yang et al. overexpressed circRNAs in organoids but evaluated their proliferation rate only by observing their morphological changes [36]. In the present study, in addition to recording the morphology of apoptotic organoids under carboplatin stress, we also calculated the exact ratio of surviving and apoptotic cells by fluorescent staining and assessed their proliferative capacity by Ki67 immunohistochemical staining, all of which were helpful in comprehensively demonstrating the effect of circRNAs on the organoids and had a greater preclinical value.

Molecular sponges of circRNAs have been widely reported as the most dominant way to regulate downstream mRNA expression [37, 38]. In the present study, we searched for possible candidate downstream mRNAs of circRAD23B by RNA-seq and found that YBX1 presented the largest differential fold change after knockdown of circRAD23B, which is a reported tumour promoter and chemoresistance inducer in OC [39–41]. Therefore, we hypothesised that circRAD23B functions by regulating YBX1. In our study, hsa-miR-1287-5p downregulated YBX1 by directly binding to its 3’ UTR, while circRAD23B indirectly upregulated YBX1 through the adsorption of hsa-miR-1287-5p, resulting in accelerated tumour proliferation and increased carboplatin resistance. YBX1, an extremely important transcription factor and RNA-binding protein, is involved in the regulation of various tumours [42]. For instance, circFAT1(e2) inhibits gastric cancer progression by targeting miR-548 g in the cytoplasm and interacting with YBX1 in the nucleus [43]. Several recent studies have shown that YBX1 acts as a circRNA-binding protein. CircRNA-SORE binds to YBX1 and enhances protein stability [44]. Another circRNA ACTN4 recruits YBX1 to activate transcription [45], while circIPO7 induces YBX1 nuclear localization [46]. Our results showed that circRNAs regulate YBX1 by acting as molecular sponges, providing directions for an in-depth study of the regulatory network of YBX1.

## Conclusions

This study showed that circRAD23B promotes proliferation and carboplatin resistance in OC cells and organoids. Mechanistically, circRAD23B abrogated the negative regulation of YBX1 by adsorbing miR-1287-5p, thereby increasing the protein level of YBX1. Thus, our study contributes to a better understanding of the molecular mechanisms underlying the development of OC and drug resistance and provides a promising therapeutic approach for circRNA-targeted OC treatment.

### Electronic supplementary material

Below is the link to the electronic supplementary material.

Supplementary Material 1

## Data Availability

The data used to support the findings of this study are available from the corresponding author upon request.

## Abbreviations

circRNAs: Circular RNAs
HE: Haematoxylin-eosin
OC: Ovarian cancer
PDOs: Patient-derived organoids
SD: Standard deviation
YBX1: Y-box binding protein 1
