# Supplementary material for: CircRAD23B promotes proliferation and carboplatin resistance in ovarian cancer cell lines and organoids
Source: Cancer Cell Int. 2024 Jan 25;24:42. doi: 10.1186/s12935-024-03228-1 (PMC10811902; doi:10.1186/s12935-024-03228-1)
Supplement: Supplementary file 1 — Supplementary Material 1 [file 12935_2024_3228_MOESM1_ESM.docx]

**Table S1. RNA-seq of circRAD23B knockdown vs negative control in HO8910 cell**

| **Gene** | **logFC** | **pvalue** | **adj.P.Val** |
| --- | --- | --- | --- |
| YBX1 | -3.938198854 | 0.033907412 | 0.285693594 |
| ALKAL2 | -2.469131497 | 1.06E-05 | 0.135374751 |
| TMED6 | -2.380839579 | 0.018936312 | 0.258305105 |
| DMBT1 | -2.309464644 | 0.039248547 | 0.297296625 |
| OLA1P1 | -2.233191747 | 0.000284064 | 0.245233149 |
| RPS3AP49 | -2.200013189 | 0.019984833 | 0.261393186 |
| RPL39P38 | -2.11370709 | 0.016910417 | 0.252979603 |
| LKAAEAR1 | -2.105415534 | 0.040702882 | 0.298346874 |
| ACTG1P17 | -2.053002286 | 0.009294397 | 0.246674558 |
| YWHAZP3 | -2.048120028 | 0.044425882 | 0.303326586 |
| NAALAD2 | -2.013537386 | 0.000185668 | 0.245233149 |
| RPL21P119 | -1.987536549 | 0.001509635 | 0.245233149 |
| RAB1C | -1.987536549 | 0.00373725 | 0.245233149 |
| PGK1P2 | -1.983512271 | 0.000549834 | 0.245233149 |
| ABCA11P | -1.973989221 | 0.000100485 | 0.245233149 |
| PLSCR3 | -1.899858414 | 0.009341374 | 0.246674558 |
| PLIN1 | -1.878344996 | 0.003373594 | 0.245233149 |
| AEBP1 | -1.872371027 | 0.019068268 | 0.258305105 |
| RPS19P1 | -1.862005667 | 0.000944644 | 0.245233149 |
| SLCO1C1 | -1.836732626 | 0.045385076 | 0.305812375 |
| ARNT2 | -1.817246351 | 0.002148016 | 0.245233149 |
| NFATC4 | -1.812180279 | 0.00070715 | 0.245233149 |
| MIR5689HG | -1.801373119 | 0.021413563 | 0.265533101 |
| PPP1R32 | -1.792549049 | 0.001070382 | 0.245233149 |
| DDX47 | -1.790563231 | 0.039418006 | 0.297296625 |
| SLC25A5P5 | -1.761512581 | 0.000217413 | 0.245233149 |
| SENP5 | -1.725132145 | 0.009649995 | 0.246674558 |
| MTND5P11 | -1.690930855 | 0.00137165 | 0.245233149 |
| MINAR1 | -1.666973001 | 0.041626102 | 0.299253725 |
| PHKG1 | -1.659517063 | 0.009928916 | 0.246674558 |
| CTSW | -1.640655888 | 0.027658961 | 0.271022365 |
| DRD4 | -1.623331129 | 0.014919202 | 0.248991994 |
| TRPM5 | -1.612359588 | 0.024927007 | 0.267779893 |
| PKP1 | -1.597561549 | 0.019458563 | 0.259366893 |
| SH2D6 | -1.56652508 | 0.033690402 | 0.285566268 |
| RPL7AP50 | -1.557463471 | 0.002569942 | 0.245233149 |
| FOXD4 | -1.533346523 | 0.007261646 | 0.246674558 |
| SLC35C1 | -1.525590784 | 0.007415022 | 0.246674558 |
| RPL17P36 | -1.512879674 | 0.010664468 | 0.247201229 |
| LEKR1 | -1.511134125 | 0.036811075 | 0.292843415 |
| LINC01671 | -1.497506593 | 0.029615458 | 0.27680409 |
| ICA1L | -1.483768063 | 0.042567602 | 0.300245672 |
| PIK3R1 | -1.476704855 | 0.03729953 | 0.293450446 |
| PIK3CB | -1.469737329 | 0.012338748 | 0.247201229 |
| ZMAT3 | -1.463238714 | 0.008275586 | 0.246674558 |
| MUC5B | -1.459215715 | 0.026855623 | 0.269918972 |
| RPGRIP1 | -1.459215715 | 0.026249656 | 0.269918972 |
| DENND10P1 | -1.445668387 | 0.045851671 | 0.305868813 |
| C4BPA | -1.419319341 | 0.0115232 | 0.247201229 |
| LRRC26 | -1.408390835 | 0.015707598 | 0.251064863 |
| PDPK2P | -1.395000689 | 0.001077996 | 0.245233149 |
| LINC01572 | -1.392540846 | 0.003224931 | 0.245233149 |
| CBX1 | -1.39122493 | 0.044113223 | 0.303139447 |
| AVL9 | -1.386939568 | 0.004281541 | 0.245233149 |
| PLSCR4 | -1.385329226 | 0.003727135 | 0.245233149 |
| RNF32 | -1.376851428 | 0.004134075 | 0.245233149 |
| LINC01568 | -1.320869882 | 0.040157175 | 0.298191463 |
| ZNF192P1 | -1.317518661 | 0.018333478 | 0.257232389 |
| FKBP1C | -1.315834251 | 0.043498705 | 0.301327772 |
| ATP6V1G2 | -1.303776381 | 0.038331512 | 0.294518379 |
| SERPINF1 | -1.280349222 | 0.039807229 | 0.298191463 |
| RAD23B | -1.27510022 | 0.02970654 | 0.27680409 |
| MTCO3P12 | -1.271597256 | 0.025275874 | 0.268315103 |
| LSM11 | -1.268630061 | 0.005138459 | 0.245233149 |
| CNOT6 | -1.246209265 | 0.003277798 | 0.245233149 |
| SLC36A1 | -1.227233506 | 0.027342322 | 0.270593975 |
| SBK3 | -1.224526678 | 0.002732202 | 0.245233149 |
| SNORA33 | -1.216956227 | 0.00435912 | 0.245233149 |
| ARHGAP6 | -1.213160298 | 0.049697099 | 0.310969416 |
| PMS2P10 | -1.210820348 | 0.045038264 | 0.304635601 |
| TRIM59 | -1.206600941 | 0.007824532 | 0.246674558 |
| MPV17L | -1.202113841 | 0.018792888 | 0.258305105 |
| ERP29P1 | -1.200013189 | 0.014567075 | 0.248991994 |
| MYLPF | -1.194982139 | 0.030171162 | 0.27680409 |
| USB1 | -1.182995716 | 0.008801294 | 0.246674558 |
| NHLH1 | -1.17655008 | 0.017365178 | 0.253528601 |
| SDCBP | -1.163428703 | 0.007407129 | 0.246674558 |
| CASS4 | -1.16289282 | 0.02804062 | 0.271262124 |
| NHP2P1 | -1.16139708 | 0.047887679 | 0.307069857 |
| IREB2 | -1.160900319 | 0.01770056 | 0.254361199 |
| NCBP2 | -1.155466681 | 0.007678118 | 0.246674558 |
| KIAA1549 | -1.145335221 | 0.011181479 | 0.247201229 |
| ADAM8 | -1.140422605 | 0.01487459 | 0.248991994 |
| MTND4P12 | -1.139299221 | 0.00417334 | 0.245233149 |
| CKS1B | -1.131253233 | 0.031262851 | 0.280971041 |
| ADD3 | -1.129159385 | 0.01427659 | 0.247823115 |
| AGPS | -1.128747773 | 0.027942654 | 0.271262124 |
| MRPS17 | -1.12868319 | 0.039867586 | 0.298191463 |
| CCDC177 | -1.119512774 | 0.013339267 | 0.247403837 |
| SNORA66 | -1.115075223 | 0.007187358 | 0.246674558 |
| LRRC15 | -1.11370709 | 0.003673146 | 0.245233149 |
| PFN1P2 | -1.11370709 | 0.021926057 | 0.265533101 |
| RPL10AP2 | -1.107501864 | 0.016191532 | 0.251064863 |
| LINC00641 | -1.102757716 | 0.036713639 | 0.292795265 |
| C2orf76 | -1.098431031 | 0.033019007 | 0.283330918 |
| SPNS1 | -1.098394995 | 0.025538379 | 0.269161078 |
| SRI | -1.092386268 | 0.042219306 | 0.299436981 |
| FAM91A1 | -1.091590917 | 0.003442705 | 0.245233149 |
| BTBD7 | -1.090927502 | 0.016073058 | 0.251064863 |
| NPM1P24 | -1.088871945 | 0.013997351 | 0.247522772 |
| PTGS1 | -1.086175218 | 0.03853111 | 0.295343727 |
| LANCL3 | -1.084038755 | 0.012230871 | 0.247201229 |
| ELK3 | -1.082339827 | 0.03392143 | 0.285693594 |
| KRT5 | -1.076696653 | 0.047870521 | 0.307069857 |
| MARCHF3 | -1.076418436 | 0.020393748 | 0.263474061 |
| CMTM4 | -1.074827669 | 0.02465552 | 0.267779893 |
| CALU | -1.07091002 | 0.031857164 | 0.282939305 |
| ARPC3P1 | -1.069240715 | 0.028579303 | 0.273377354 |
| ANKRD46 | -1.057573521 | 0.008859124 | 0.246674558 |
| SIRT4 | -1.054636012 | 0.019839765 | 0.261322128 |
| ATF7IP2 | -1.054349259 | 0.047915538 | 0.307069857 |
| RAP2A | -1.052990056 | 0.03727985 | 0.293450446 |
| LRP12 | -1.048851878 | 0.013045366 | 0.247201229 |
| BMT2 | -1.045756159 | 0.009289582 | 0.246674558 |
| QSOX2 | -1.042650017 | 0.026380955 | 0.269918972 |
| SPOPL | -1.038794115 | 0.015013311 | 0.248991994 |
| RPL4P3 | -1.038204247 | 0.030655962 | 0.278936308 |
| EEF1A1P9 | -1.03777146 | 0.014255777 | 0.247823115 |
| POLQ | -1.036420409 | 0.007609075 | 0.246674558 |
| KPNA4 | -1.035130149 | 0.006623774 | 0.246674558 |
| HOTAIRM1 | -1.029981451 | 0.047043718 | 0.306825594 |
| RPL31P49 | -1.028488798 | 0.001496215 | 0.245233149 |
| DLG1 | -1.026902519 | 0.04383383 | 0.302609853 |
| STEAP4 | -1.025767572 | 0.005281536 | 0.245233149 |
| KLLN | -1.02317495 | 0.026308948 | 0.269918972 |
| ACER2 | -1.019398328 | 0.011855949 | 0.247201229 |
| SHC3 | -1.018370576 | 0.005410628 | 0.245233149 |
| SMURF2 | -1.016028924 | 0.030303698 | 0.2772104 |
| HOOK3 | -1.013938989 | 0.014976242 | 0.248991994 |
| ZNF280C | -1.006808305 | 0.045471801 | 0.305868813 |
| PPTC7 | -1.002380301 | 0.012880998 | 0.247201229 |
| LSMEM1 | -1.002130447 | 0.005427886 | 0.245233149 |
| ZNF721 | 1.00213422 | 0.004099782 | 0.245233149 |
| ZCCHC3 | 1.003929394 | 0.006644343 | 0.246674558 |
| CRYAB | 1.007416813 | 0.035310406 | 0.2887927 |
| ZDHHC16 | 1.009516168 | 0.016867409 | 0.252979603 |
| PAGE2 | 1.016691871 | 0.010373895 | 0.246674558 |
| CCDC9 | 1.0167422 | 0.000222481 | 0.245233149 |
| ZNF766 | 1.019275057 | 0.046400032 | 0.305868813 |
| PODXL2 | 1.020569004 | 0.00164256 | 0.245233149 |
| ECE1 | 1.026706358 | 0.009582983 | 0.246674558 |
| TLK2 | 1.027984533 | 0.014064221 | 0.247522772 |
| XKR5 | 1.028353201 | 0.019917896 | 0.261393186 |
| SYTL2 | 1.029403581 | 0.007090424 | 0.246674558 |
| GRIPAP1 | 1.036338757 | 0.008087742 | 0.246674558 |
| DKK3 | 1.037838706 | 0.014672584 | 0.248991994 |
| WBP4 | 1.038021343 | 0.017284192 | 0.253528601 |
| ZNF860 | 1.03806865 | 0.010775941 | 0.247201229 |
| STAC | 1.040808819 | 0.002955018 | 0.245233149 |
| UNC13A | 1.042268662 | 0.046321885 | 0.305868813 |
| TRIOBP | 1.044348138 | 0.004392776 | 0.245233149 |
| TGFB3 | 1.055698331 | 0.027635937 | 0.271022365 |
| TTC21A | 1.064069487 | 0.034051141 | 0.285693594 |
| MYO15B | 1.064069487 | 0.041744821 | 0.299253725 |
| ANG | 1.066691571 | 0.007732772 | 0.246674558 |
| HOGA1 | 1.06792128 | 0.002693663 | 0.245233149 |
| FOXD3 | 1.072101379 | 0.005366327 | 0.245233149 |
| MST1P2 | 1.075288584 | 0.007349985 | 0.246674558 |
| SNX18P3 | 1.082652447 | 0.012465332 | 0.247201229 |
| HIRA | 1.084646013 | 0.017464622 | 0.253528601 |
| TYMP | 1.093664743 | 0.049057627 | 0.310513686 |
| PPP1R14BP3 | 1.095105955 | 0.000915581 | 0.245233149 |
| ZNF184 | 1.095963936 | 0.00922339 | 0.246674558 |
| MT2A | 1.099580291 | 0.028487405 | 0.273274392 |
| SLC16A4 | 1.100563108 | 0.025332496 | 0.268315103 |
| ACOT1 | 1.101113403 | 0.003829879 | 0.245233149 |
| CCDC189 | 1.1019622 | 0.042425111 | 0.299728737 |
| PCK2 | 1.102190544 | 0.008718117 | 0.246674558 |
| LINC00346 | 1.107183411 | 0.005441279 | 0.245233149 |
| SETBP1 | 1.107339181 | 0.001287832 | 0.245233149 |
| DHDH | 1.107597524 | 0.002826882 | 0.245233149 |
| MESD | 1.108502853 | 0.013727418 | 0.247522772 |
| PAQR6 | 1.110213639 | 0.02155445 | 0.265533101 |
| NINL | 1.118603831 | 0.006717205 | 0.246674558 |
| NUDT3 | 1.122930192 | 0.001325658 | 0.245233149 |
| EDNRA | 1.124177899 | 0.013212972 | 0.247403837 |
| ZNF284 | 1.12507373 | 0.001583371 | 0.245233149 |
| TSPOAP1 | 1.125329801 | 0.022263957 | 0.265533101 |
| HCN4 | 1.129295867 | 0.032601283 | 0.283330918 |
| LRRN2 | 1.135598625 | 0.01485368 | 0.248991994 |
| LINC00337 | 1.136226944 | 0.020378571 | 0.263474061 |
| ADTRP | 1.143235926 | 0.010685708 | 0.247201229 |
| IFI27L1 | 1.146826022 | 0.006537271 | 0.246674558 |
| LINC01145 | 1.153962727 | 0.013038932 | 0.247201229 |
| CBR3 | 1.155062612 | 0.038105078 | 0.293946175 |
| MEX3D | 1.158775665 | 0.005153211 | 0.245233149 |
| CCDC80 | 1.159123209 | 0.005927209 | 0.245233149 |
| LINC02798 | 1.160107268 | 0.008283726 | 0.246674558 |
| FAM21EP | 1.16697936 | 0.000100757 | 0.245233149 |
| ACTA2 | 1.168035778 | 0.026840508 | 0.269918972 |
| FAAHP1 | 1.169319228 | 0.043422242 | 0.301327772 |
| TNFRSF25 | 1.173321878 | 0.002391131 | 0.245233149 |
| FBXO27 | 1.179963565 | 0.045824442 | 0.305868813 |
| KATNAL2 | 1.182388453 | 0.03605554 | 0.291071933 |
| LRRC75A | 1.187094136 | 0.015733187 | 0.251064863 |
| LINC02361 | 1.190354541 | 0.020224811 | 0.263019334 |
| TBC1D10B | 1.195261804 | 0.001103996 | 0.245233149 |
| SIPA1L1 | 1.196727375 | 0.044346784 | 0.303326586 |
| ZCWPW1 | 1.201211569 | 0.002600188 | 0.245233149 |
| BICRA | 1.205304363 | 0.000312177 | 0.245233149 |
| EHD4 | 1.206112506 | 0.042082951 | 0.299253725 |
| PLEKHO1 | 1.216534806 | 0.008467802 | 0.246674558 |
| IGFBP4 | 1.224143887 | 0.009463243 | 0.246674558 |
| NES | 1.224740698 | 0.011603196 | 0.247201229 |
| LINC01816 | 1.231569941 | 0.005794907 | 0.245233149 |
| PGM5 | 1.235546203 | 0.043894485 | 0.302609853 |
| LINC01135 | 1.243765141 | 0.000836645 | 0.245233149 |
| LUNAR1 | 1.2457121 | 0.00670618 | 0.246674558 |
| MEOX1 | 1.250137479 | 0.049480698 | 0.310513686 |
| HABP4 | 1.250217695 | 0.006445441 | 0.246191788 |
| ZNF765 | 1.250232917 | 0.000338659 | 0.245233149 |
| LINC02289 | 1.25054529 | 0.007469983 | 0.246674558 |
| GANAB | 1.252267097 | 0.034083892 | 0.285693594 |
| ABHD17AP3 | 1.253285459 | 0.040513537 | 0.298346874 |
| ZNF57 | 1.259259428 | 0.045425376 | 0.305868813 |
| RRBP1 | 1.26059165 | 0.015906079 | 0.251064863 |
| SULF1 | 1.264092618 | 0.04334846 | 0.301276499 |
| SYNE3 | 1.268272717 | 0.003113437 | 0.245233149 |
| CLIP2 | 1.282884794 | 0.003882781 | 0.245233149 |
| GAS1 | 1.283171837 | 0.009349288 | 0.246674558 |
| C2orf92 | 1.286470227 | 0.035556811 | 0.290064361 |
| SPINK5 | 1.290093456 | 0.000539058 | 0.245233149 |
| PLCB2 | 1.29117659 | 0.000671201 | 0.245233149 |
| EIF4EP2 | 1.307186957 | 0.016455562 | 0.251064863 |
| OR2A9P | 1.309927126 | 0.013317786 | 0.247403837 |
| PLEK2 | 1.3214462 | 0.048752356 | 0.309947272 |
| ANKRD1 | 1.326759294 | 0.00982967 | 0.246674558 |
| C1GALT1C1L | 1.327416266 | 0.005776697 | 0.245233149 |
| R3HCC1 | 1.329582525 | 0.009697445 | 0.246674558 |
| SCNN1D | 1.346225635 | 0.040817869 | 0.298346874 |
| ADAM15 | 1.349461419 | 0.005639074 | 0.245233149 |
| IFT27 | 1.353162927 | 0.016446748 | 0.251064863 |
| ZNF227 | 1.363707446 | 0.001241484 | 0.245233149 |
| GEMIN8P4 | 1.375030248 | 0.013886868 | 0.247522772 |
| THTPA | 1.377861753 | 0.000502626 | 0.245233149 |
| ALPG | 1.380398754 | 0.044430266 | 0.303326586 |
| CHST14 | 1.382492385 | 0.00245994 | 0.245233149 |
| CASP7 | 1.391242055 | 0.005386156 | 0.245233149 |
| CPED1 | 1.396788169 | 0.030781155 | 0.279367316 |
| TGM1 | 1.397417539 | 0.001475456 | 0.245233149 |
| CCDC144B | 1.404580541 | 0.00061662 | 0.245233149 |
| CFTR | 1.404580541 | 0.020869989 | 0.265347009 |
| LGR5 | 1.406429332 | 0.036657224 | 0.292795265 |
| SLC7A8 | 1.406651635 | 0.003319206 | 0.245233149 |
| LMTK3 | 1.411401619 | 0.00056719 | 0.245233149 |
| CCT6B | 1.412956485 | 0.005532211 | 0.245233149 |
| ZBTB12 | 1.41869816 | 0.000222142 | 0.245233149 |
| TACSTD2 | 1.418800194 | 0.003405483 | 0.245233149 |
| PPARG | 1.422508301 | 0.009481575 | 0.246674558 |
| ERAP2 | 1.427960199 | 0.040796394 | 0.298346874 |
| ZCCHC2 | 1.433847093 | 0.025167459 | 0.268211893 |
| YPEL1 | 1.451738016 | 0.045111571 | 0.304635601 |
| CCDC96 | 1.468795567 | 0.000363975 | 0.245233149 |
| NAALADL1 | 1.476569259 | 0.012517147 | 0.247201229 |
| TPM4 | 1.507408634 | 0.032043929 | 0.283276876 |
| CTSH | 1.536364369 | 0.00179629 | 0.245233149 |
| VSIG10L2 | 1.546244199 | 0.038133452 | 0.293946175 |
| H2BC5 | 1.558196913 | 0.004336114 | 0.245233149 |
| RGS19 | 1.569518585 | 0.009550269 | 0.246674558 |
| LINC01006 | 1.589192472 | 0.002312923 | 0.245233149 |
| SPTBN5 | 1.591451982 | 0.001268434 | 0.245233149 |
| ZNF501 | 1.597425952 | 0.023243869 | 0.267779893 |
| LINC02767 | 1.610137661 | 0.001094612 | 0.245233149 |
| LDB3 | 1.620889061 | 0.030179034 | 0.27680409 |
| LAP3 | 1.622570443 | 0.031375266 | 0.28138937 |
| LYPD6 | 1.622712448 | 0.011206642 | 0.247201229 |
| PIK3CD | 1.623031151 | 0.006473676 | 0.246191788 |
| ZNF695 | 1.630233303 | 0.005375876 | 0.245233149 |
| B3GNT5 | 1.635918358 | 0.014272203 | 0.247823115 |
| MT1DP | 1.64809365 | 0.019110656 | 0.258305105 |
| MYO1G | 1.648753771 | 0.021297014 | 0.265533101 |
| DYNLRB2 | 1.654067619 | 0.003377815 | 0.245233149 |
| ARL2BP | 1.670051599 | 0.000957954 | 0.245233149 |
| RN7SL1 | 1.672549653 | 0.028540214 | 0.273377354 |
| TERT | 1.682740945 | 0.026057965 | 0.269918972 |
| WNT5B | 1.702108886 | 0.004513413 | 0.245233149 |
| ODF3B | 1.703823971 | 0.0441915 | 0.303326586 |
| DLK2 | 1.732831019 | 0.011274387 | 0.247201229 |
| TNRC6C | 1.747829656 | 0.026630439 | 0.269918972 |
| PIWIL2 | 1.76115884 | 0.0033915 | 0.245233149 |
| ANGPTL2 | 1.798314119 | 0.011302368 | 0.247201229 |
| B3GNT3 | 1.812044681 | 0.045624879 | 0.305868813 |
| KLF15 | 1.826446181 | 0.005818509 | 0.245233149 |
| SCGB2B2 | 1.84308115 | 0.048044384 | 0.307345918 |
| CTAGE4 | 1.856628478 | 0.002501994 | 0.245233149 |
| FOXCUT | 1.884924777 | 0.015756344 | 0.251064863 |
| KIAA1217 | 1.89373653 | 0.011664618 | 0.247201229 |
| ZNF572 | 1.907296176 | 0.0157723 | 0.251064863 |
| HIC1 | 1.950390515 | 0.024851258 | 0.267779893 |
| LRRC56 | 1.969251691 | 0.007478113 | 0.246674558 |
| SPSB3 | 1.9927148 | 0.00176388 | 0.245233149 |
| C1QTNF2 | 2.038609695 | 0.001058399 | 0.245233149 |
| SYCE3 | 2.079020899 | 0.000678215 | 0.245233149 |
| FAM117A | 2.115877965 | 0.037878989 | 0.293860241 |
| ZNF836 | 2.119772816 | 0.012278418 | 0.247201229 |
| AGAP10P | 2.125746786 | 0.008987254 | 0.246674558 |
| MDK | 2.135509194 | 0.042985032 | 0.300925757 |
| TRIML2 | 2.158033965 | 0.002038742 | 0.245233149 |
| DPY19L2P2 | 2.198858739 | 0.000604193 | 0.245233149 |
| PLK5 | 2.199877593 | 0.004092517 | 0.245233149 |
| EGFEM1P | 2.271548556 | 0.023111963 | 0.267779893 |
| ANO7L1 | 2.338223425 | 0.026440292 | 0.269918972 |
| C17orf113 | 2.357461925 | 0.016368477 | 0.251064863 |
| CCDC159 | 2.394646949 | 0.033045144 | 0.283330918 |
| DDO | 2.409743377 | 0.048404257 | 0.308784947 |
| ZG16 | 2.642348804 | 0.005115026 | 0.245233149 |
| CD22 | 2.776248421 | 0.004148345 | 0.245233149 |
| OGFR | 2.847659468 | 0.003977063 | 0.245233149 |
| SYNGR4 | 2.869872289 | 0.007232914 | 0.246674558 |
| GRIN3B | 2.987400953 | 0.014389331 | 0.248535936 |
| CARD16 | 3.129737666 | 0.021590872 | 0.265533101 |
